# Taking the PubChem web sketcher to the next level

**DOI:** 10.1186/1758-2946-5-S1-P20

**Published:** 2013-03-22

**Authors:** Wolf D Ihlenfeldt

**Affiliations:** 1 Xemistry GmbH, Königstein, 61462, Germany

## 

The input of chemical structures in the context of Web applications is a well-known interface problem in chemical information processing. Currently, this is typically achieved by using either Java applets, or platform- and browser-specific plug-ins - both approaches require installation of custom software components which may not be possible in many use cases. Pure client-side JavaScript solutions have recently been presented, but these require the latest generation of browsers, and are significantly limited in the functionality they can provide.

The first popular Web-bases structure sketcher to break out of these moulds was the PubChem structure input tool. It is based on a server-based image streaming model, backward-compatible down to IE6 in a fully browser- and platform-agnostic fashion, and usable without any installation of client software. This software has been in continuous development since it was first deployed about five years ago. The latest version, which still maintains exceptional legacy browser compatibility, has evolved into an information hub which can directly query dozens of Internet chemistry databases. It now supports advanced HTML5 features, such as drag&drop of structure data files and pasting of ChemDraw or ISIS/Draw data on the clipboard. An innovative operation mode for use on touchscreens with limited resolution and no mouse, and voice control for structure input on small devices or for handicapped users are other recently added features.

